# Expression Profiling and Transcriptional Coordination of Canonical Wnt Signaling Pathway Genes (FZD2, DVL2, and AXIN1) in Chronic Venous Insufficiency

**DOI:** 10.3390/biomedicines14081725

**Published:** 2026-07-31

**Authors:** Jinan Abugharsa, Özlem Balcıoğlu, Barçın Özcem, Mahmut Cerkez Ergoren, Aya Badeea Ismail, Selma Yilmaz

**Affiliations:** 1 Department of Medical Biology and Genetics, Faculty of Medicine, Near East University, Nicosia 99138, Cyprus; 2 Department of Cardiovascular Surgery, Faculty of Medicine, Near East University, Nicosia 99138, Cyprus; 3 Medical Genetics Diagnostic Laboratory, Near East University Hospital, Nicosia 99138, Cyprus; 4 Department of Molecular Medicine, Faculty of Medicine, Near East University, Nicosia 99138, Cyprus

**Keywords:** chronic venous insufficiency, gene expression, Wnt pathway, FZD2, DVL2, AXIN1

## Abstract

**Background/Objectives:** Chronic venous insufficiency (CVI) is a progressive vascular disorder characterized by sustained venous hypertension leading to structural and functional alterations of the venous wall. Despite its clinical significance, the molecular mechanisms driving venous remodeling remain incompletely understood. The Wnt signaling pathway has emerged as an important regulator of vascular homeostasis and remodeling; however, the expression and coordination of its core components in chronic venous insufficiency (CVI)-affected tissue have not been directly investigated. This study aimed to analyze mRNA expression of FZD2, DVL2, and AXIN1 in great saphenous vein specimens. In addition to gene expression analysis at the individual level, an investigation of co-regulatory networks was also performed to provide insight into Wnt cascade regulation in the varicose and healthy vein tissues. **Methods:** This single-center, retrospective observational study was conducted on 60 great saphenous vein specimens (32 CVI patients and 28 healthy controls) using re-al-time quantitative PCR. **Results:** The findings indicate that individual mRNA expression levels of FZD2, DVL2, and AXIN1 remained stable between varicose vein and healthy control tissues, but there were significant changes in transcriptional coordination among these genes in varicose vein tissues, especially enhanced co-expression between AXIN1 and DVL2. All three co-regulated genes exhibited moderate and generally comparable positive correlations in healthy controls: FZD2 with DVL2 (r = 0.526, *p*-value = 0.0069), DVL2 with AXIN1 (r = 0.486, *p*-value = 0.0161), and AXIN1 with FZD2 (r = 0.593, *p*-value = 0.0014). While in varicose vein tissues, DVL2-AXIN1 showed the highest correlation (r = 0.722, *p*-value < 0.0001), FZD2-DVL2 (r = 0.517, *p*-value = 0.0096) and AXIN1-FZD2 (r = 0.480, *p*-value = 0.0084) remained comparable to the healthy tissues. **Conclusions:** This finding indicates that the involvement of FZD2, DVL2, and AXIN1 in CVI may be driven by altered transcriptional coordination rather than by differential individual gene expression, which may contribute to the molecular pathology of chronic venous insufficiency.

## 1. Introduction

**Figure 1:**
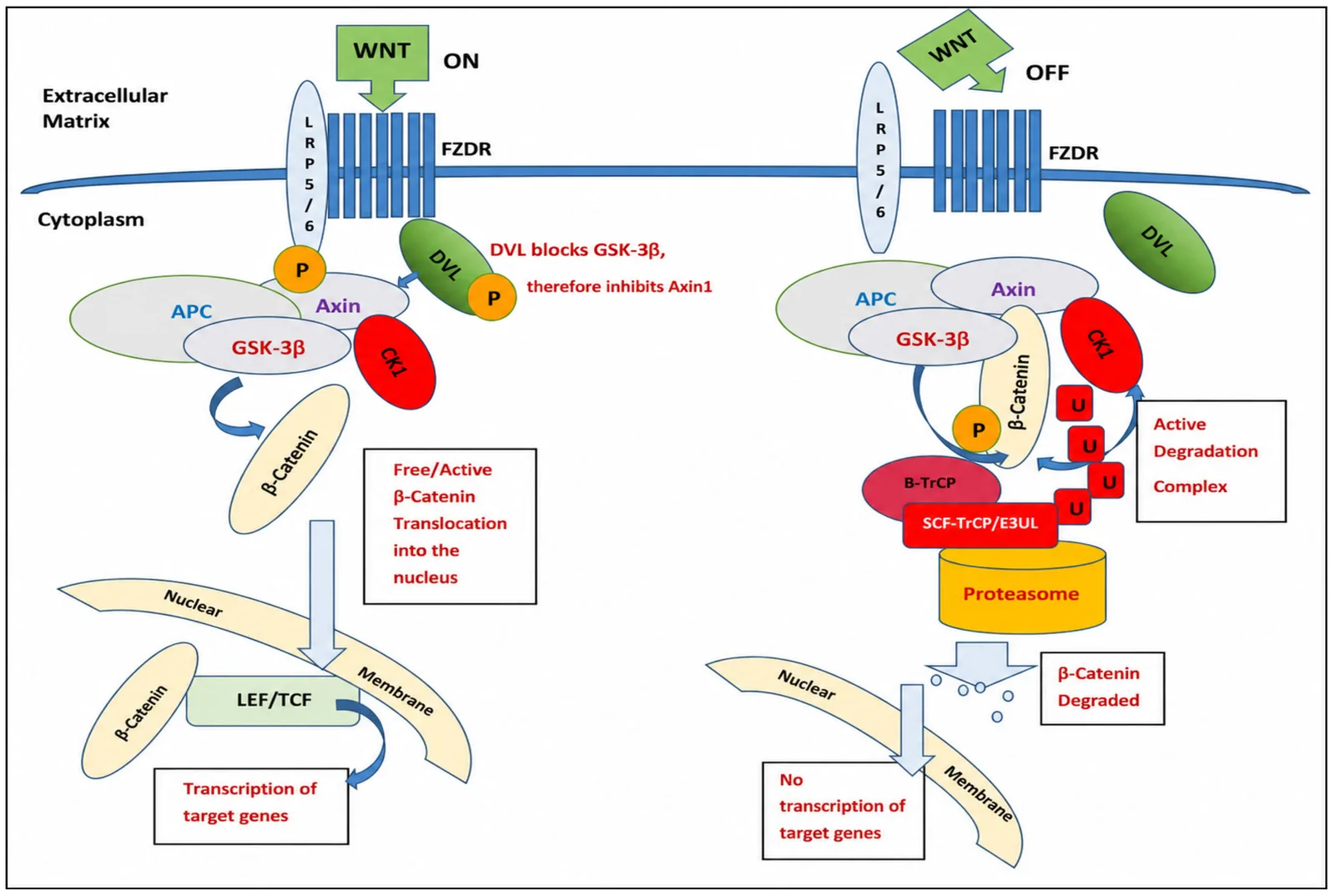
Overview of the canonical Wnt/β-catenin signaling pathway. Schematic representation of the Wnt/β-catenin signal transduction pathway. (Left) In the “On” state, the frizzled and LRP5/6 receptor complex binds Wnt and recruits the disheveled (DVL) protein to the plasma membrane. As a result, several components of the β-catenin destruction complex are recruited to the membrane, preventing β-catenin phosphorylation. As a result, this protein can now concentrate in the cytoplasm before translocating to the nucleus, where it can interact with transcription factors and increase the transcription of Wnt target genes, such as cyclin D1, c-myc, and AXIN2. (Right) In the “Off” state, β-catenin is bound in a so-called β-catenin destruction complex that includes glycogen synthase kinase 3 β (GSK3 β), AXIN, adenomatous polyposis coli (APC), and casein kinase-1 (CK-1). The kinases in this complex phosphorylate β-catenin, which is then targeted for degradation by the ubiquitin–proteasome system. SCFβ-TrCP E3 ubiquitin ligases degrade and break down β-catenin by identifying substrates with β-TrCP proteins. T-cell-specific transcription factor (TCF) and lymphoid enhancer-binding factor (LEF) collaborate to activate the target gene. Created by S. Yilmaz.

Chronic venous insufficiency (CVI) causes morphological and functional problems in the venous system. CVI is a progressive vascular disorder, characterized by venous hypertension, valvular incompetence, and structural remodeling of the venous wall, most commonly manifesting as varicose veins, lower limb edema, and venous ulceration [1,2,3]. Studies indicate that approximately 25% of the population has varicose veins, with up to 5% experiencing more severe problems [4]. Beyond its considerable prevalence, CVI carries serious complications if left untreated. One of the most significant risks is the development of superficial venous thrombosis (SVT), particularly in the femoropopliteal veins, which increases the risk of deep vein thrombosis (DVT) and pulmonary thromboembolism, a potentially fatal condition [5,6]. Risk factors are well documented, including aging, female sex, pregnancy, prolonged standing, and a history of thrombosis [7]. Venous insufficiency is classified according to the clinical, etiologic, anatomical, and pathophysiological classification system (CEAP), which includes seven major classes, ranging from C0 to C6 [8]. Although diagnostic methods have improved, including color duplex ultrasonography (CDU) [9,10], there is no treatment for reversing the progression of CVI [11]. One major cause for this constraint is the lack of understanding of disease mechanisms. On the cellular level, chronic inflammation, endothelial dysfunction, vascular smooth muscle (VSM) cell phenotypic switching, and extracellular matrix (ECM) destruction contribute to the development of CVI [1,12]. This entire set of processes underlies the structural breakdown of the venous wall and the development of venous hypertension. Recent studies implicated dysregulated Wnt signaling pathways as critical factors involved in this process. The Wnt signaling pathway is an evolutionarily conserved signaling system involved in essential physiological functions, such as proliferation, differentiation, and apoptosis [13,14,15]. Wnt dysregulation has been found in cardiovascular disorders pathophysiologically related to CVI, including atherosclerosis, heart fibrosis, and vascular remodeling [16,17,18,19,20,21]. The selected Wnt genes, including Frizzled 2 (FZD2), Disheveled 2 (DVL2), and Axis Inhibition Protein 1 (AXIN1), are key players in the Wnt cascade. FZD2, DVL2, and AXIN1 were selected because they represent three distinct functional tiers of the canonical Wnt pathway. These genes serve as receptors, intracellular signal transducers, and negative regulators of the canonical Wnt/β-catenin pathway, respectively [22,23,24,25]. Their coordinated selection enabled evaluation of transcriptional coordination across different levels of the pathway, while their dysregulation contributes to endothelial dysfunction and vascular remodeling. The function of Wnt signaling occurs through three major branches, namely, the canonical Wnt/β-catenin, the non-canonical pathways of Planar Cell Polarity (PCP), and the Wnt/Ca^2+^ pathways. The Wnt/Planar Cell Polarity (PCP) pathway acts through Rho-family GTPases to regulate cytoskeletal organization and cell polarity. The Wnt/Ca^2+^ pathway triggers intracellular calcium release and downstream effectors, such as CaMKII and PKC. Notably, FZD2 and DVL2 are shared components across all three pathways, with their downstream actions varying according to cellular conditions [13,14,15]. In the canonical Wnt pathway, DVL2 activation by FZD2 inhibits the AXIN1-containing destruction complex (AXIN1·APC·GSK-3β·CK1), inhibiting β-catenin phosphorylation and degradation, and enabling nuclear translocation to trigger LEF/TCF-dependent transcription (Figure 1) [26].

Given that DVL2 and AXIN1 play directly opposing functional roles in this regulatory switch, their transcriptional co-expression could indicate coordinated pathway activity important to CVI pathophysiology [18,25,26]. Despite growing molecular evidence supporting a role for Wnt signaling in the pathogenesis of CVI, these genes have not been thoroughly investigated in the context of venous insufficiency. With regard to the development of CVI, a previous study demonstrated that Wnt3a, a canonical ligand, was aberrantly expressed, whereas no observable difference was found for the non-canonical Wnt5a [3]. Following up on the results of previous studies, the present study analyzed the expression profiles of upstream key canonical Wnt signaling components, such as FZD2, DVL2, and AXIN1, in great saphenous vein (GSV) tissues of patient and healthy groups using quantitative real-time PCR. In addition to individual gene expression analysis, the present study also evaluated the transcriptional co-regulation between these genes in both groups. This approach aimed to provide insight into the transcriptional co-regulation of canonical Wnt signaling components underlying chronic venous insufficiency pathology beyond conventional differential expression analysis.

## 2. Materials and Methods

### 2.1. Study Design and Vascular Material Collection

The present study was designed as a single-center, retrospective observational study using previously collected data. Although this was a single-center study, the patient population at Near East University Hospital (NEUH) is highly heterogeneous, and patients represent a multicultural demographic structure of the island with diverse ethnic backgrounds, thereby increasing the genetic diversity of the study population. A total of 60 great saphenous vein samples were obtained from patients who underwent varicose vein and bypass surgeries at Near East University Hospital. At the Near East University Hospital’s Radiology Department, the GSV samples underwent a comprehensive assessment of their venous system using Doppler ultrasound equipment (Doppler ultrasound examinations were performed at Near East University Hospital, Nicosia, Cyprus, using either a LOGIQ S6 ultrasound system (GE HealthCare, Chicago, IL, USA) or an ACUSON S2000 ultrasound system (Siemens Healthineers, Erlangen, Germany). The samples were separated into two groups: the patient group, comprising 32 abnormal GSVs classified as C2 according to the CEAP classification system, corresponding to varicose veins, which indicated they met the criteria for venous insufficiency, and the control group, comprising 28 healthy GSVs. Meanwhile, the control group participants were also patients requiring coronary artery bypass surgery who had no record of varicose veins or abnormal great saphenous veins as determined by Doppler ultrasonography. Control GSV segments were obtained from unused great saphenous vein grafts collected during coronary artery bypass grafting (CABG) procedures, representing a widely accepted and ethically appropriate source of non-varicose venous tissue for comparative molecular studies. This approach is commonly used in CVI research and has been routinely adopted at Near East University Hospital. Subsequently, the individuals engaged in comprehensive meetings with cardiovascular surgeons in the cardiovascular surgery department at Near East University Hospital in order to deliberate about the procedure of mini-phlebectomy. Through mini-phlebectomy surgery, a biological sample consisting of a segment of GSV that encompassed all vascular layers was extracted from each participant and subsequently transferred into individual, clearly labeled sterile tubes. The collected material was appropriately labeled and immediately frozen and stored at −80 °C until it was ready for further processing to investigate gene expression and ensure its preservation until the collection process was complete. This strategy is a commonly and ethically accepted method for acquiring healthy venous tissue in CVI research, as the vein is typically removed during the surgery with no additional intervention. The Near East University Scientific Review Board approved the experimental investigation (Approval ID: NEU/2025/133-1962; Approval Date: 30 April 2025), which strictly adhered to ethical standards.

### 2.2. RNA Isolation and cDNA Synthesis

RNA was isolated in strict compliance with (Cortex Science kit, Adana, Turkey). A Nanodrop spectrophotometer (Thermo Scientific, Pittsburgh, PA, USA) was used to measure RNA purity. RNA purity was assessed based on the A260/A280 absorbance ratio, with values between 1.8 and 2.0 considered acceptable. Subsequently, complementary DNA (cDNA) synthesis was performed using (Hibrigen kit, Gebze, Kocaeli, Turkey).

### 2.3. Primer Optimization, Gradient PCR, and Gel Electrophoresis

The first step beyond the fundamental components of PCR is to combine the samples containing the target sequence with primers. To analyze FZD2, DVL2, and AXIN1, specific forward and reverse primers were designed using the Primer-BLAST (NCBI) tool (National Center for Biotechnology Information, Bethesda, MD, USA; https://www.ncbi.nlm.nih.gov/tools/primer-blast/, accessed on 20 April 2025), (see Supplementary Materials). Primer specificity was confirmed by gradient PCR and agarose gel electrophoresis using expected amplicon sizes.

Gradient polymerase chain reaction (PCR) was performed to determine the optimal annealing temperature for the amplification of the target genes. Several annealing temperatures ranging from 56 to 60 °C were tested to determine the best conditions for primer amplification. The specific nature of primers and their amplification efficiency were analyzed independently under the same experimental conditions. Primers were synthesized at a stock concentration of 10 μM and utilized at a final concentration of 0.2 μM. According to the optimization results, an annealing temperature of 60 °C was set for FZD2, DVL2, and AXIN1 for subsequent analyses. These optimized conditions improved the reliability and reproducibility of the results.

Gel electrophoresis was performed using a 3% agarose gel run in 1× Tris-Borate–EDTA (TBE) buffer to analyze the polymerase chain reaction (PCR) products.

### 2.4. Gene Expression Analysis

Gene expression levels were measured using a quantitative real-time PCR (qPCR) detection system over 35 PCR cycles. qPCR enables the quantification of mRNA content in samples rather than only detecting the presence or absence of a specific target sequence. As a series of independent experiments, the optimized conditions were replicated, including primer concentrations (10 μM) and working volume (0.2 μM), along with annealing temperatures of 60 °C for each gene. Utilizing a 2× SYBR Green qPCR kit (Hibrigen, Gebze, Kocaeli, Turkey), 4 samples were analyzed in duplicate, along with 2 negative control samples. The lack of signals in the negative control samples reflects an absence of primer dimers. All double strands exhibited fluorescent emission that was undoubtedly generated from the specified sequence. Samples containing the target sequence were combined with primers using a 2x SYBR Green qPCR master mix (Hibrigen, Turkey) kit.

During the annealing stage, the primers bind to the target sequence in cDNA. The DNA polymerase enzyme then elongates DNA sequences specifically during the extension stage, allowing for the creation of a new DNA strand. During each cycle, the dye probe binds to freshly produced double strands and emits fluorescence that the qPCR machine detects. The level of fluorescence emitted during each cycle directly correlates with the quantity of target nucleic acid in the sample. The lack of signals in the negative control samples reflects an absence of primer dimers. All double strands exhibited fluorescent emission that was undoubtedly generated from the specified sequence.

Expression levels of target genes FZD2, DVL2, and AXIN1, along with the ACTB housekeeping gene, were detected by real-time qPCR under the thermal cycling conditions.

### 2.5. Statistical Analysis

The statistical analysis for this study was conducted using the SPSS program (Statistical Package for the Social Sciences 25.0, SPSS Inc., Chicago, IL, USA), and GraphPad Prism software, version 11.0.0 (84). Correlation analyses were conducted to examine the relationships between ACTB and target genes in both control and patient groups across a total of 60 specimens. To evaluate gene expression, cycle threshold (Ct) values and relative gene expression were obtained across all samples using the 2^−∆∆Ct^ method and are presented as fold changes. Cycle threshold (Ct) values in qPCR measure the amplification cycles needed for a fluorescent signal to cross a set threshold. The Ct values are inversely proportional to the starting target concentration, with lower values indicating high target abundance. Spearman’s rank analysis between ACTB and the three target genes in each of the two groups was conducted on Ct values at a significance level of *p* < 0.05. All error bars indicate the standard deviation (SD) of the mean Ct values of the biological replicate samples. The Shapiro–Wilk tests were used to determine the distribution’s normality. The Mann–Whitney U test was used to compare ΔCt values (Ct target − Ct housekeeping), as the data were not normally distributed. The target genes’ fold expression levels were measured using the 2^−ΔΔCt^ technique. Because of the variability found across several datasets, the results are presented as the median (IQR). All bars represent median fold change values of biological replicate samples. Spearman’s rank correlation test was also used to assess correlation, and a base transcriptional network analysis was conducted to evaluate gene co-regulation patterns for each gene pair in both healthy control and patient groups.

The study’s findings were considered statistically significant (*p* < 0.05) based on observed differences and correlations.

## 3. Results

### 3.1. Population Study Description

The study population consisted of patients diagnosed with varicose veins and a control group without the condition. This study was conducted at NEUH (Near East University Hospital) and included 60 samples divided into two groups: the patient group (32 individuals) and the control group (28 individuals). A large majority of participants in the control group (64.3%) were male, and 35.7% were female. In contrast, males and females were equally represented (50% male, 50% female) in the patient group. The observed differences in age and sex distribution between the patient and control groups reflect the clinical source of the control specimens. Healthy control tissue was obtained from surplus coronary artery bypass grafting (CABG) material, which is typically collected from older individuals and predominantly male patients. Therefore, complete demographic matching was not feasible. The age and gender of the study population are presented in Table 1.

Quantitative real-time PCR was used to evaluate gene expression over 35 cycles, with a consistent annealing temperature of 60 °C for the target genes (FZD2, DVL2, and AXIN1) and ACTB (β-actin) in both control and patient groups across 60 specimens. Statistical analyses of average cycle threshold (Ct) values were performed to evaluate the correlations between the housekeeping gene (ACTB) and the target genes (FZD2, DVL2, and AXIN1) in both groups.

### 3.2. Correlation of the Expression Level Between Examined Genes and the Housekeeping Gene

**Figure 2:**
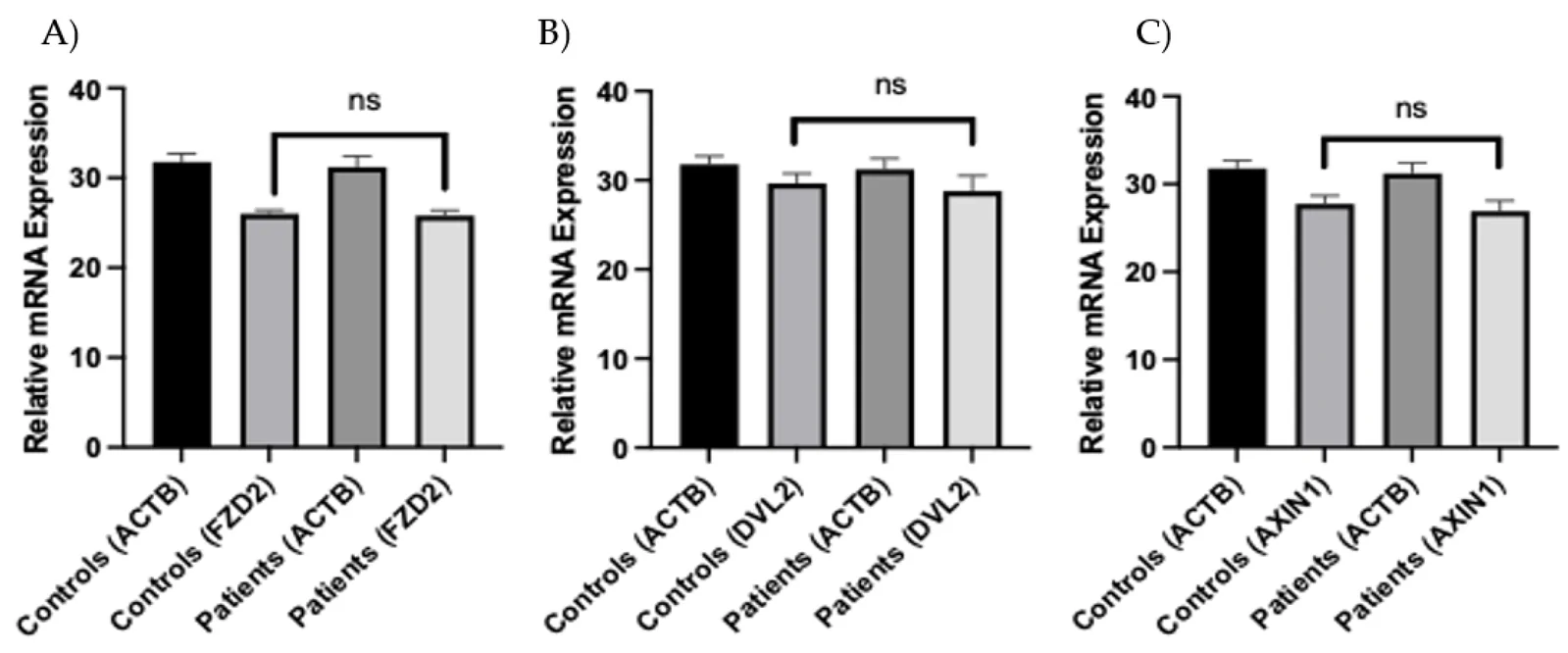
Relative mRNA expression levels of FZD2, DVL2, and AXIN1 in control and patient groups. All error bars indicate the SD of the mean Ct values of the biological replicate samples. Spearman’s correlation analysis between ACTB and target genes showed the following *p*-values: (**A**) ACTB vs. FZD2 (control *p*-value = 0.0879, n = 27; patient *p*-value = 0.6841, n = 31); (**B**) ACTB vs. DVL2 (control *p*-value = 0.3000, n = 25; patient *p*-value = 0.7478, n = 26); (**C**) ACTB vs. AXIN1 (control *p*-value = 0.1172, n = 26; patient *p*-value = 0.3198, n = 31). ns = not statistically significant (*p* > 0.05).

Correlations among gene expression levels were assessed using Spearman’s rank correlation on Ct values at a significance level of *p*-value < 0.05. For the verification of reference gene stability, Spearman’s correlation analysis between ACTB and the three target genes in each of the two groups was conducted. In the control group, the correlations between ACTB and FZD2 (r = 0.3347, *p*-value = 0.0879), between ACTB and DVL2 (r = 0.2159, *p*-value = 0.3000), and between ACTB and AXIN1 (r = 0.3149, *p*-value = 0.1172) were evaluated. In the patient group, the correlations between ACTB and FZD2 (r = −0.077, *p*-value = 0.6841), between ACTB and DVL2 (r = −0.06771, *p*-value = 0.7478), and between ACTB and AXIN1 (r = 0.1880, *p*-value = 0.3198) were examined as well. None of the correlations between ACTB and the three target genes reached the significance threshold value (*p*-value > 0.05), suggesting that ACTB expression was not significantly associated with FZD2, DVL2, and AXIN1. The Ct values (mean ± SD) for the target genes were as follows: FZD2, 26.011 ± 0.388 (n = 27) in controls and 25.806 ± 0.589 (n = 31) in patients; DVL2, 29.665 ± 1.085 (n = 25) in controls and 28.791 ± 1.734 (n = 26) in patients; and AXIN1, 27.752 ± 0.946 (n = 26) in controls and 26.926 ± 1.202 (n = 31) in patients. The corresponding *p*-values are indicated in Figure 2.

### 3.3. Fold Change in Expression Levels Between Target Gene Groups

The relative mRNA expression levels of FZD2, DVL2, and AXIN1 were compared between varicose vein and control subjects. Statistical comparisons were performed using the Mann–Whitney U test on ΔCt values (Ct target − Ct housekeeping) because data were not normally distributed. The fold expression levels of the target genes were assessed using the 2^−ΔΔCt^ method. Results are shown as median (IQR) due to variability observed in multiple datasets. The relative expression of FZD2 was comparable between the control and patient groups, with a *p*-value = 0.2098. In the healthy group, FZD2 showed moderate dispersion in normal venous tissue (median: 1.097; IQR: 0.8257–1.4681; n = 27).

In varicose vein tissue, the relative expression of FZD2 was comparable between control and patient groups (median: 1.003; IQR: 0.5021–1.4032; n = 30). The coefficient of variation was higher in the patient group (CV: 62.05%) compared to controls (CV: 43.12%). These findings suggest that no evidence of differential FZD2 expression was detected in the studied condition.

Similarly, the DVL2 expression was broadly equivalent in patients compared to controls, with a *p*-value = 0.2293. DVL2 in controls showed marked variability (median: 0.9587; IQR: 0.7265–1.2476; CV: 116.57%; n = 25). In varicose tissues, DVL2 expression showed a median similar to controls but higher variability (median: 1.514; IQR: 0.4630–3.8349; CV: 141.97%; n = 25), indicating high variability in both groups, with greater dispersion in patients.

**Figure 3:**
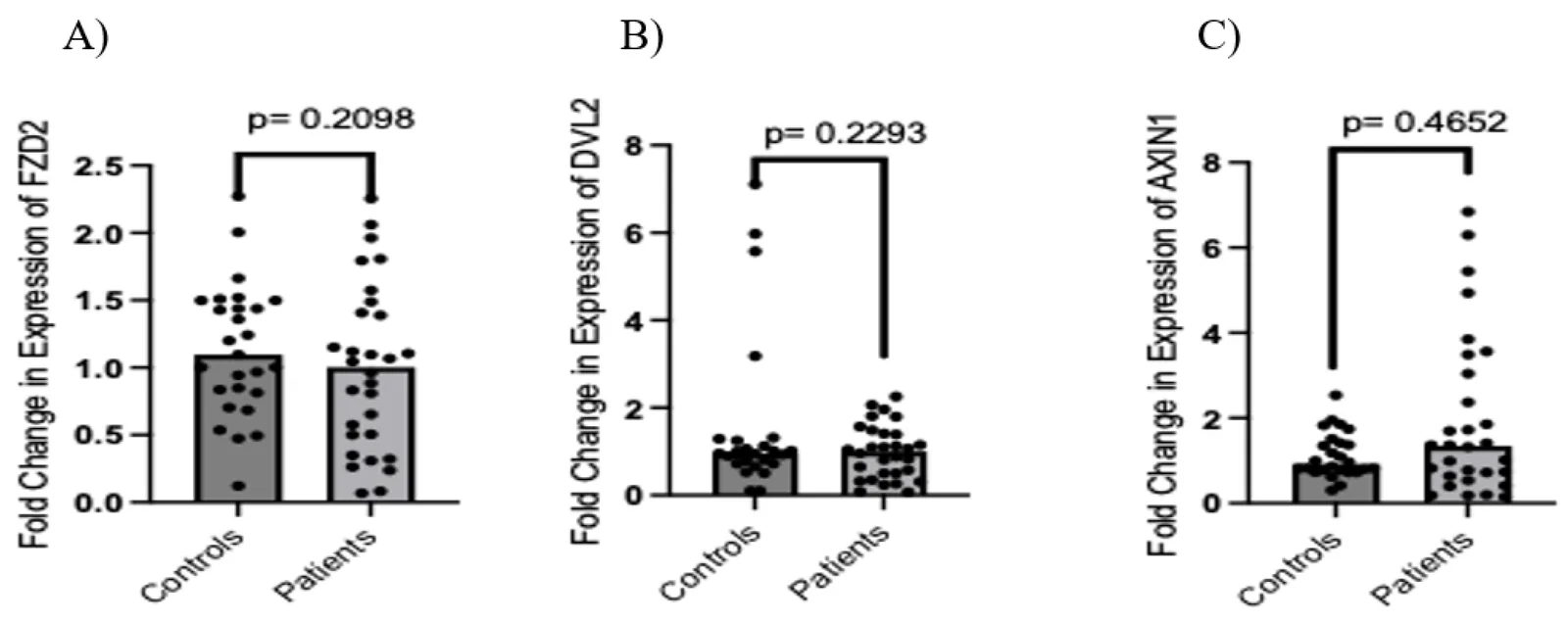
Fold change in expression levels of (**A**) FZD2, (**B**) DVL2, and (**C**) AXIN1 in controls and patients. Fold change values were calculated using the 2^−ΔΔCt^ method. All bars represent median fold change values of biological replicate samples. Exact *p*-values are shown above each comparison. Sample sizes: (**A**) n = 27 controls, n = 30 patients; (**B**) n = 25 controls, n = 25 patients; (**C**) n = 26 controls, n = 30 patients (*p* > 0.05).

Likewise, AXIN1 expression was comparable in patients compared to controls, with a *p*-value = 0.4652. In healthy tissues, AXIN1 showed intermediate variability (median: 0.9209; IQR: 0.7760–1.4062; CV: 47.77%; n = 26). In comparison, AXIN1 expression indicated elevated variability in varicose veins (median: 1.342; IQR: 0.6603–2.8724; CV: 97.36%; n = 30). As a result, the comparative fold change in expression levels of FZD2, DVL2, and AXIN1 showed no statistically significant differences between control and patient groups (*p* > 0.05) (Figure 3).

### 3.4. Coordinated Expression Analysis Among Target Genes in Varicose and Healthy Tissues

**Figure 4:**
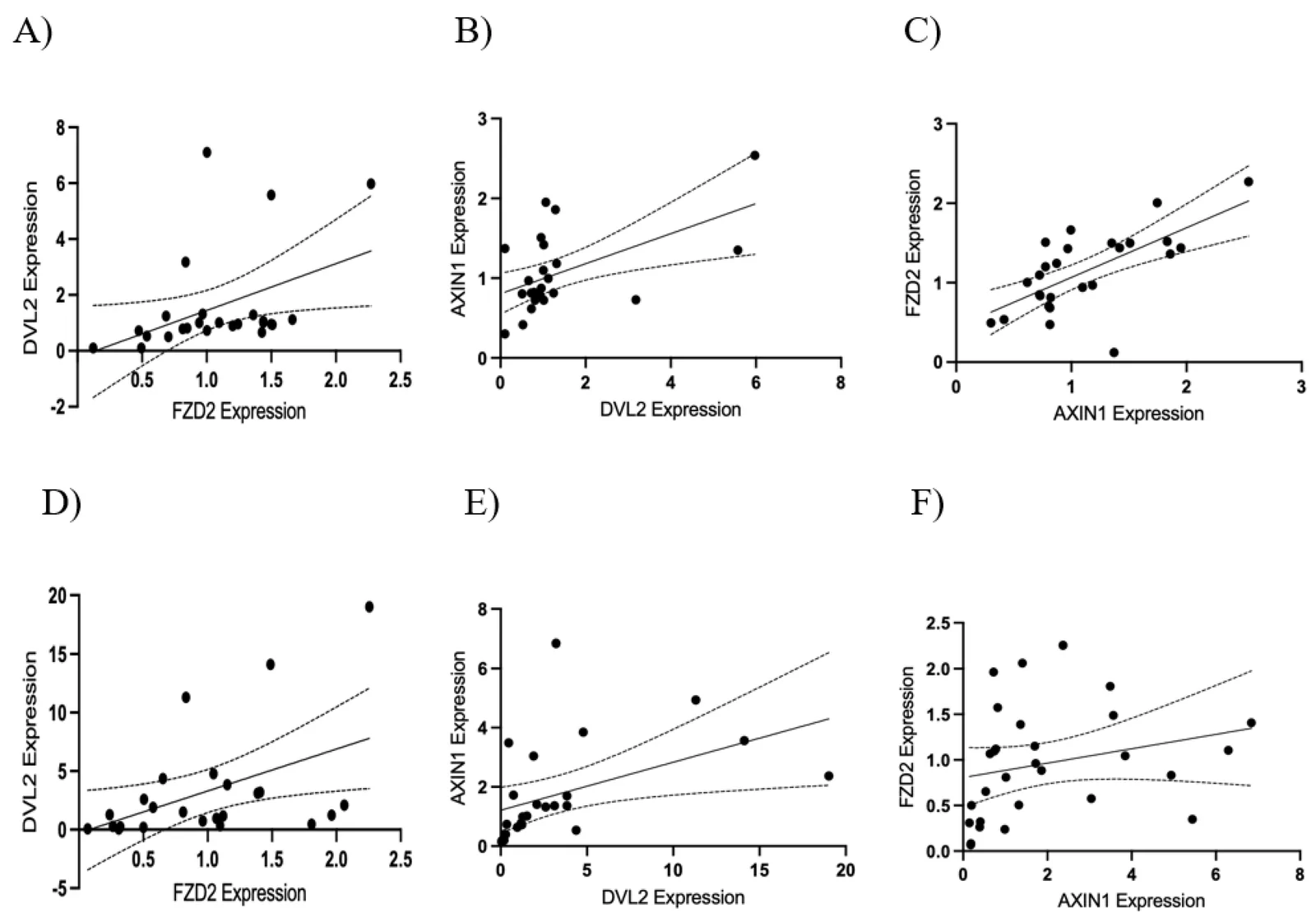
Spearman’s correlation analysis of fold change in expression levels (2^_ΔΔCt^) between FZD2, DVL2, and AXIN1 in healthy controls and varicose vein patients. In healthy tissues, significant positive correlations were observed between (**A**) FZD2 and DVL2 (*p*-value = 0.0069, n = 25), (**B**) DVL2 and AXIN1 (*p*-value = 0.0161, n = 24), and (**C**) AXIN1 and FZD2 (*p*-value = 0.0014, n = 26). In varicose vein tissues, significant positive correlations were also observed between (**D**) FZD2 and DVL2 (*p*-value = 0.0096, n = 24), (**E**) DVL2 and AXIN1 (*p*-value < 0.0001, n = 25), and (**F**) AXIN1 and FZD2 (*p*-value = 0.0084, n = 29).

Spearman’s rank correlation analysis based on relative fold-change expression values (2^−ΔΔCt^) demonstrated significant co-expression among FZD2, DVL2, and AXIN1 in both healthy controls and varicose vein patients. The strength and hierarchy of gene-to-gene relationships differed, which suggests a shift in Wnt pathway transcriptional coordination in CVI. In healthy tissues, the three gene pairs showed moderate and relatively comparable correlations: FZD2 with DVL2 (r = 0.526, *p*-value = 0.0069, n = 25), DVL2 with AXIN1 (r = 0.486, *p*-value = 0.0161, n = 24), and AXIN1 with FZD2 (r = 0.593, *p*-value = 0.0014, n = 26) (Figure 4). In varicose vein tissues, all three correlations remained statistically significant, but the DVL2–AXIN1 relationship was markedly stronger (r = 0.722, *p*-value < 0.0001, n = 25), while FZD2–DVL2 (r = 0.517, *p*-value = 0.0096, n = 24) and AXIN1–FZD2 (r = 0.480, *p*-value = 0.0084, n = 29) remained at moderate levels similar to those observed in controls (Figure 4).

## 4. Discussion

Chronic venous insufficiency is a progressive vascular disorder characterized by sustained venous hypertension, valvular incompetence, and chronic inflammation, which collectively drive structural deterioration of the venous wall [1,27]. The molecular mechanisms underlying this deterioration remain incompletely understood, and this gap is a primary reason why no disease-modifying treatment currently exists [11,28]. In this context, the Wnt/Frizzled axis has attracted growing interest for its well-established roles in vascular development, smooth muscle cell regulation, and pathological cardiovascular remodeling [29]. In particular, the canonical Wnt/β-catenin pathway governs a broad range of cellular processes, including proliferation, survival, and extracellular matrix turnover that are directly relevant to the structural changes observed in CVI-affected venous tissue [30,31,32], and understanding the functional organization of Wnt signaling pathways is essential.

To interpret these findings, it is essential to understand the functional organization of Wnt signaling pathways. In the canonical pathway, Wnt ligand engagement of FZD receptors and LRP5/6 co-receptors triggers activation of DVL proteins and subsequent inhibition of the β-catenin destruction complex, in which AXIN1 serves as the central scaffold alongside APC and GSK-3β. The result is cytoplasmic β-catenin accumulation and nuclear translocation, driving TCF/LEF-dependent transcription of genes involved in proliferation, inflammation, and matrix remodeling [25,26,30,31,32] (Figure 1). In parallel, non-canonical Wnt signal transduction pathways are important for the organization of the cytoskeleton, intracellular calcium levels, and cell polarity. Several studies have shown that the non-canonical Wnt signaling pathway modulates the canonical Wnt signaling pathway under physiological and pathological conditions [33,34]. Abnormalities in WNT signaling have been previously shown to contribute to vascular smooth muscle cell proliferation, endothelial activation, and fibrotic remodeling in various cardiovascular diseases, such as atherosclerosis and myocardial injury [35,36,37,38]. Within this framework, FZD2, DVL2, and AXIN1 represent key functional components acting as receptor, signal transducer, and scaffold/negative regulator, respectively; dysregulation in these genes could theoretically affect venous wall remodeling and endothelial dysfunction in CVI.

Evidence from cardiovascular research can also give further context regarding the roles of these genes in venous diseases. FZD2 was found to be highly upregulated in atherosclerotic plaques and abnormal remodeling of blood vessels [18]; DVL2 inhibits vascular smooth muscle cell proliferation and migration in arterial injury [23]. Similarly, AXIN1 modulates cardiomyocyte injury under ischemia–reperfusion conditions [24] and hypoxic stress, consistent with its established role as a stress-responsive scaffold within the canonical Wnt destruction complex [37]. These findings collectively highlight the importance of Wnt signaling components in vascular disease contexts.

In the present study, no significant difference in the expression levels of mRNA for FZD2, DVL2, and AXIN1 was observed between CVI patients and healthy subjects. This finding does not imply non-involvement of the pathway but instead reflects the maintenance of stability in the transcription of these genes [39]. Since canonical Wnt signaling depends on β-catenin stabilization and nuclear translocation rather than solely on transcriptional changes in individual pathway components [25,30,32], stable mRNA expression levels do not necessarily exclude Wnt pathway modulation. Importantly, the findings of this research are in line with previous data showing that the signaling activity in the canonical Wnt pathway is mainly post-translational regulation and not alterations in mRNA expression [39]. Post-translational modification, polymerization, auto-inhibition of AXIN1, protein phosphorylation, and subcellular redistribution have been reported as mechanisms for activating the Wnt/β-catenin signaling pathway without altering the expression levels of the mRNAs encoding key proteins [40,41,42]. Therefore, the stable mRNA expression levels of FZD2, DVL2, and AXIN1 in CVI-affected venous tissue do not exclude Wnt pathway modulation.

Despite individual gene expression levels remaining relatively stable, the results of the correlation analysis were more meaningful. In healthy controls, all three co-regulated genes showed moderate and broadly similar positive correlations: FZD2 with DVL2 (r = 0.526, *p*-value = 0.0069), DVL2 with AXIN1 (r = 0.486, *p*-value = 0.0161), and AXIN1 with FZD2 (r = 0.593, *p*-value = 0.0014). No single gene pair was clearly related to strengthening; the overall pattern reflects the balanced functional interdependence expected of a receptor, a transducer, and a negative regulatory scaffold operating together within the canonical LRP5/6-FZD-DVL-AXIN regulatory network [40,42]. In varicose vein tissue, this balance was disrupted in a specific and informative way. While all three correlations remained statistically significant, the DVL2–AXIN1 axis became substantially altered in coordination (r = 0.722, *p*-value < 0.0001), clearly exceeding both FZD2–DVL2 (r = 0.517, *p*-value = 0.0096) and AXIN1–FZD2 (r = 0.480, *p*-value = 0.0084). Mechanistically, the selective strengthening of the DVL2–AXIN1 co-expression in varicose vein tissue may reflect their direct molecular relationship within the canonical pathway, in which DVL2 recruits AXIN1 to the plasma membrane via their shared DIX domains, sequestering it away from the cytoplasmic destruction complex and thereby redirecting GSK3β activity away from β-catenin phosphorylation [18]. The strong DVL2–AXIN1 association observed in varicose vein tissues may indicate a particular axis between the central signal transducer and the destruction complex scaffold without a corresponding shift in receptor level correlations. These results suggest altered transcriptional coordination may therefore represent a pathway-level response to the chronic venous hypertension and inflammatory stress characteristic of CVI [1,12,43]. Collectively, the presence of significant correlations among FZD2, DVL2, and AXIN1 indicates that the involvement of Wnt signaling in CVI is not primarily driven by changes in individual gene expression levels but rather by alterations in transcriptional coordination within the pathway. This supports the concept that CVI-related molecular changes may occur at the network level rather than through isolated gene dysregulation [44]. Thus, traditional differential expression analysis alone might not adequately reflect pathway-level changes associated with CVI, but a network-based approach could offer additional biological understanding.

Certain biological mechanisms could explain this molecular pattern. As previously reported in chronic venous insufficiency [3], pathological stresses such as inflammation, hypoxia, and endothelial injury can increase the expression of the canonical ligand Wnt3a. Increased Wnt3a levels can cause pathological vascular remodeling by enhancing β-catenin signaling in vascular cells. In contrast, core pathway components such as FZD2, DVL2, and AXIN1 remain transcriptionally stable, most likely because these proteins are constitutively available at basal levels and can transduce increased ligand signals without the necessity for additional gene expression [25,42]. This is consistent with the canonical Wnt signaling mechanism, which is primarily regulated by protein–protein interactions and post-translational modifications rather than transcriptional changes [25,40,41,42]. When Wnt3a binds to FZD receptors, DVL2 is recruited and activated at the plasma membrane. This promotes AXIN1 relocation and destabilizes the β-catenin destruction complex, allowing β-catenin stabilization and nuclear translocation [25,40,41]. Furthermore, reduced activity of extracellular Wnt inhibitors, such as secreted frizzled-related proteins (sFRPs), may improve ligand responsiveness without affecting receptor or scaffold gene expression [18,29]. These mechanisms suggest that Wnt pathway activation in chronic venous insufficiency is primarily mediated by dynamic regulation of pre-existing signaling proteins rather than transcriptional upregulation of FZD2, DVL2, or AXIN1.

In chronic vascular diseases (such as atherosclerosis or venous insufficiency), gene expression of the ligand Wnt3a is frequently upregulated, while AXIN1, DVL2, and FZD receptors do not change significantly. This occurs because Wnt signaling is primarily influenced by extracellular ligands and intracellular protein activation, rather than changes in the transcription of its core machinery [3,25,41,42]. Specifically, this molecular phenomenon is driven by specific biological factors. In chronic vascular diseases, vascular stress, hypoxia, or inflammation causes adjacent cells (such as macrophages and myointimal cells) to overexpress the Wnt3a ligand. Consequently, excessive Wnt3a causes pathological changes, such as the transdifferentiation of Vascular smooth muscle cells (VSMCs) into osteoblast-like cells (vascular calcification) and increased vessel permeability [1,2]. Regarding the components of cellular signaling, these include FZD receptors, DVL2, and AXIN1. In healthy states, vascular tissues have a consistent, baseline presence of these genes and proteins. During chronic disease, however, the cell does not need to increase the transcription of these receptor or scaffold genes because the current (basal) levels are adequate to bind the overabundant Wnt3a and transmit signals [1,2,3]. The Wnt pathway is primarily regulated by protein–protein interactions and post-translational modifications (such as phosphorylation), not by gene transcription [1,2,3,4]. Furthermore, chronic vascular stress frequently reduces the expression of inhibitory Wnt regulators, such as secreted frizzled-related proteins (sFRPs). When these natural “brakes” are removed, the baseline levels of FZD and DVL2 become hyperactive, causing cells to be highly responsive to Wnt3a even when receptor or scaffold gene expression remains constant [1,3].

In contrast, these results differ from cases of several arterial and cardiac diseases, wherein the expression of FZD2 increases during cardiac hypertrophy and is upregulated in atherosclerotic plaques [18,35], while DVL2 is activated in vascular smooth muscle cell injury and atrial remodeling [23], and AXIN1 transcription is markedly elevated in cardiac ischemia–reperfusion injury [24]. In CVI, none of these changes were found, as all three genes exhibited relatively stable mRNA expression. It seems that since similar transcriptional changes were not observed in CVI, the Wnt pathway’s role may differ between cardiac and venous diseases. Taken together, FZD2, DVL2, and AXIN1 showed no significant transcriptional differences between CVI patients and healthy controls, indicating stable mRNA abundance in varicose vein tissue. However, the selective strengthening of the DVL2–AXIN1 relationship in varicose vein tissue, while FZD2’s correlations remained comparable between groups, suggests that conventional differential expression analysis alone may not fully capture Wnt pathway involvement in CVI. Wnt signaling regulates matrix metalloproteinase expression and extracellular matrix turnover in vascular tissue [45], and since progressive matrix degradation drives venous wall weakening and varicose vein formation [46], this selective DVL2–AXIN1 coordination may contribute to that remodeling process. These findings highlight the value of network-based approaches for understanding Wnt signaling in CVI pathophysiology.

Finally, several limitations should be considered when interpreting the findings. The limitations of this study include age mismatch and insufficient sample size from a single center. Large sample sizes and more heterogeneous populations would improve statistical power. In addition, there could have been variations in gene expression due to the presence of heterogeneity in terms of clinical features among the CVI cases that may influence the Wnt signaling pathway. Another limitation is that only the level of mRNA was determined, without the measurement of proteins or even β-catenin activity. Future studies may include additional studies on Wnt signaling in varicose veins.

## 5. Conclusions

The present study demonstrates that, despite unchanged individual mRNA expression levels of FZD2, DVL2, and AXIN1, these genes exhibit a significant positive correlation in mRNA expression that was observed for each gene pair in both healthy controls and patients. This includes the DVL2-AXIN1 co-expression, although the correlation might be stronger in the patients based on the *p*-value. In chronic vascular problems, including atherosclerosis or arterial stiffness, pathological stress upregulates paracrine Wnt3a ligands, mainly via invading immune cells. However, intracellular components (AXIN 1 and DVL2) and receptors (FZD) remain statically expressed because the pathway is activated post-translationally, relying on pre-existing baseline protein pools rather than new transcription to drive disease. These findings suggest that certain genes involved in chronic venous insufficiency may influence post-translational activation processes after Wnt3a binds to the FZD receptor. This process recruits and polymerizes DVL2 protein without upregulating DVL2 or FZD gene expression. AXIN1 is a scaffold protein for the β-catenin destruction complex that is either sequestered to the cell membrane or degraded during Wnt3a pathway activation, resulting in stable baseline mRNA levels despite increased pathway activity. Additionally, upregulation in Wnt3a may also use the existing pool of FZD receptors, eliminating the need for new receptor transcription. Given the well-established role of canonical Wnt signaling in vascular remodeling, endothelial homeostasis, and smooth muscle cell regulation, these coordination changes may reflect subtle molecular disturbances contributing to venous wall pathology. The results highlight the utility of network-based transcriptional analyses for revealing pathway-level alterations in chronic venous insufficiency. Further studies integrating protein-level analyses and functional investigations are needed to better elucidate the role of Wnt signaling in chronic venous insufficiency pathophysiology.

## Figures and Tables

**Table 1:** Age and gender of the study population.

Analysis Categories	Controls (n = 28)	Patients (n = 32)
Female	35.7% (n = 10)	50% (n = 16)
Male	64.3% (n = 18)	50% (n = 16)
Mean (Years)	59	51
Median (Years)	64	50
Age Range (Years)	67	41
**Age Group (Years)**		
20–39	15.4% (n = 4)	30% (n = 9)
40–59	15.4% (n = 4)	30% (n = 9)
60–79	69.2% (n = 18)	33.3% (n = 10)
80–99	0% (n = 0)	6.7% (n = 2)

## Data Availability

Data are available upon request from the corresponding author.

## Supplementary Materials

The following supporting information can be downloaded at https://www.mdpi.com/article/10.3390/biomedicines14081725/s1, Table S1. The primers designed for target genes; Table S2. The conditions used for real-time qPCR target gene expression analysis.

## Abbreviations

The following abbreviations are used in this manuscript:

Wnt	Wingless
FZD2	Frizzled 2
DVL2	Disheveled 2
AXIN1	Axis inhibitor1
ACTB	β-actin
LRP	Lipoprotein receptor protein
APC	Adenomatous polyposis
GSK	Glycogen synthase kinase
β-TrCP	β-Transducing repeat-containing protein
SCF	Skp1–Cullin1–F-box E3 ubiquitin ligase complex
TCF	T-cell factor
LEF	Lymphoid enhancer binding factor
PCP	Planar cell polarity
sFRPs	Secreted frizzled-related proteins
CVI	Chronic venous insufficiency
VSMC	Vascular smooth muscle cell
ECM	Extracellular matrix
VVs	Varicose veins
CEAP	Clinical, etiologic, anatomic, and pathophysiologic
DVT	Deep vein thrombosis
SVT	Superficial venous thrombosis
CDU	Color duplex ultrasonography
GSV	Great saphenous vein
NEUH	Near East University Hospital
CABG	Coronary artery bypass grafting
cDNA	Complementary DNA
PCR	Polymerase chain reaction
mRNA	Messenger ribonucleic acid
RT-qPCR	Quantitative reverse transcription polymerase chain reaction
Ct	Cycle threshold
