# Supplementary material for: Expression Profiling and Transcriptional Coordination of Canonical Wnt Signaling Pathway Genes (FZD2, DVL2, and AXIN1) in Chronic Venous Insufficiency
Source: Biomedicines. 2026 Jul 31;14(8):1725. doi: 10.3390/biomedicines14081725 (PMC13509367; doi:10.3390/biomedicines14081725)
Supplement: Supplementary file 1 [file biomedicines-14-01725-s001.zip › biomedicines-4418283-supplementary.pdf]

Supplementary Materials

Supplementary Table S1. The primers designed for target genes.

Specifies the Primers Pairs' Target Base Sequences 5'-3			Product size
FZD2	Forward	GCCCTCATGAACAAGTTCGGT	108 bp
	Reverse	GTCCTCGGAGTGGTTCTGGC	
DVL2	Forward	CAGGAGAGACAGCAGTGAGC	111 bp
	Reverse	TCGCTGGTCATGAGGGTAGA	
AXIN1	Forward	CTGTCCAGCGATGCAGACAC	150 bp
	Reverse	GTACGTGCGGGGAATGTGAG	

Supplementary Table S2. The conditions conducted for real time-qPCR target genes expression analysis.

Stage	Temperature	Time
Initial Denaturation	95°C	5 Minutes
Denaturation	95°C	10 Seconds
Annealing	60°C	15 Seconds
Extension	72°C	20 Seconds
Final Extension	72°C	2 Minutes
